# Phenotypical and Myopathological Consequences of Compound Heterozygous Missense and Nonsense Variants in *SLC18A3*

**DOI:** 10.3390/cells10123481

**Published:** 2021-12-09

**Authors:** Adela Della Marina, Annabelle Arlt, Ulrike Schara-Schmidt, Christel Depienne, Andrea Gangfuß, Heike Kölbel, Albert Sickmann, Erik Freier, Nicolai Kohlschmidt, Andreas Hentschel, Joachim Weis, Artur Czech, Anika Grüneboom, Andreas Roos

**Affiliations:** 1 Department of Pediatric Neurology, Centre for Neuromuscular Disorders, Centre for Translational Neuro- and Behavioral Sciences, University Duisburg-Essen, 45122 Essen, Germany; Ulrike.Schara-Schmidt@uk-essen.de (U.S.-S.); andrea.gangfuss@uk-essen.de (A.G.); Heike.Koelbel@uk-essen.de (H.K.); Andreas.Roos@uk-essen.de (A.R.); 2 Institute of Clinical Genetics and Tumor Genetics Bonn, 53111 Bonn, Germany; Annabelle.Arlt@ukmuenster.de (A.A.); kohlschmidt@genetik-bonn.de (N.K.); 3 Institute of Human Genetics, University Hospital Essen, University of Duisburg-Essen, 45122 Essen, Germany; christel.depienne@uk-essen.de; 4 Leibniz-Institut für Analytische Wissenschaften -ISAS- e.V., 44139 Dortmund, Germany; albert.sickmann@isas.de (A.S.); erik.freier@ruhr-uni-bochum.de (E.F.); andreas.hentschel@isas.de (A.H.); artur.czech@isas.de (A.C.); anika.grueneboom@isas.de (A.G.); 5 Institute of Neuropathology, University Hospital Aachen, RWTH-Aachen University, 52074 Aachen, Germany; JWeis@ukaachen.de; 6 Children’s Hospital of Eastern Ontario Research Institute, Ottawa, ON K1H 8L1, Canada

**Keywords:** *SLC18A1*, congenital myasthenic syndrome, vesicular acetylcholine transporter (VAChT), lipid accumulation, muscle biopsy, CARS microscopy

## Abstract

Background: Presynaptic forms of congenital myasthenic syndromes (CMS) due to pathogenic variants in *SLC18A3* impairing the synthesis and recycling of acetylcholine (ACh) have recently been described. *SLC18A3* encodes the vesicular ACh transporter (VAChT), modulating the active transport of ACh at the neuromuscular junction, and homozygous loss of VAChT leads to lethality. Methods: Exome sequencing (ES) was carried out to identify the molecular genetic cause of the disease in a 5-year-old male patient and histological, immunofluorescence as well as electron- and CARS-microscopic studies were performed to delineate the muscle pathology, which has so far only been studied in VAChT-deficient animal models. Results: ES unraveled compound heterozygous missense and nonsense variants (c.315G>A, p.Trp105* and c.1192G>C, p.Asp398His) in *SLC18A3*. Comparison with already-published cases suggests a more severe phenotype including impaired motor and cognitive development, possibly related to a more severe effect of the nonsense variant. Therapy with pyridostigmine was only partially effective while 3,4 diaminopyridine showed no effect. Microscopic investigation of the muscle biopsy revealed reduced fibre size and a significant accumulation of lipid droplets. Conclusions: We suggest that nonsense variants have a more detrimental impact on the clinical manifestation of *SLC18A3*-associated CMS. The impact of pathogenic *SLC18A3* variants on muscle fibre integrity beyond the effect of denervation is suggested by the build-up of lipid aggregates. This in turn implicates the importance of proper VAChT-mediated synthesis and recycling of ACh for lipid homeostasis in muscle cells. This hypothesis is further supported by the pathological observations obtained in previously published VAChT-animal models.

## 1. Introduction

Congenital myasthenic syndromes (CMS) are a heterogeneous group of rare genetic diseases characterized by impaired neuromuscular transmission. Clinical symptoms usually manifest in early childhood (most often within the first two years of life) and disease progression and severity varies greatly with ranges from severe “floppy infants” to the presence of minor symptoms such as ptosis and mild muscle weakness. On a general note, early onset might be associated with bulbar and respiratory symptoms, generalized fluctuating muscle hypotonia and ptosis with or without ophthalmoplegia. Undiagnosed, a proportion of neonatal onset cases develop infection-triggered critical situations with respiratory symptoms (apnoeas) resulting in hypoxic injury [1]. In adult patients, limb-girdle muscular weakness and fatigable weakness are prominent clinical features [2]. Presynaptic forms of CMS due to mutations in *SLC5A7* and *SLC18A3*, impairing the synthesis and recycling of acetylcholine (ACh), have recently been described [3,4]. The *SLC18A3* gene on chromosome 10q11.23 encodes the vesicular acetylcholine transporter (VAChT) modulating the active transport of ACh at the neuromuscular junction (NMJ). VAChT knockout in mice results in a lethal phenotype [5]. Interestingly, missense variants and whole gene deletions have been previously identified in CMS-patients, defining *SLC18A3* as a CMS-causative gene (CMS subtype 21; MIM: #617239), a molecular genetic observation in agreement with the above-mentioned function of the corresponding protein. These patients presented with features seen in presynaptic CMS forms, including ptosis, ophthalmoplegia, bulbar symptoms, fatigable weakness, decrement on low-frequency repetitive stimulation followed by a prolonged period of post-activation exhaustion, and apnoeic crises with moderate clinical improvement upon treatment with pyridostigmine (PS) [6,7]. Given that one patient additionally showed learning difficulties and left ventricular dysfunction [3], a phenotypical variability of pathogenic *SLC18A3-*mutations is likely. Along this line, a recent publication reported on a severe prenatal phenotype defined by akinesia, arthrogryposis, edema, and partial cleft palate based on a homozygous nonsense variant (c.1116C>A; p.Cys372Ter) in the *SLC18A3* gene [8]. Based on the genotype–phenotype correlation, the authors postulated that *SLC18A3* nonsense variants cause a more severe phenotype than missense variants. This assumption would be in line with previous studies showing a lethal phenotype in VAChT knockout mice [5].

Here, we present a long-term follow-up clinical description along with the first reported myopathological study of a paediatric patient presenting with compound heterozygous pathogenic variants (NM_003055:c.315G>A, p.Trp105* and c.1192G>C, p.Asp398His;) in *SLC18A3*. The benefit of PS is increasingly being reported for different CMS subtypes (depending on the underlying pathogenic variant) including ACh deficiency and glycosylation abnormalities, but only partial clinical improvement under PS treatment was observed in our patient.

## 2. Materials and Methods

Written informed consent for clinical description, inclusion of photographs of the patient in publications, genetic studies and use of the muscle biopsy for research purposes, was obtained from both patients’ parents. This study was approved by the Ethics Committee of the University of Essen (19-9011-BO). The study was conducted in accordance with the principles of the Declaration of Helsinki 1975, as revised in 1983. The data that support the findings of this study are available from the corresponding author upon request.

A biopsy of the vastus lateralis muscle was performed for diagnostic purposes. Cryostat sections (6 µm) were processed for routine histological and immunohistochemical staining and histochemical reactions including Oil Red O. Electron microscopy (EM) of ultrathin sections from glutaraldehyde-fixed, resin-embedded muscle was performed using a Philips CM10 transmission electron microscope [9].

### 2.1. Molecular Genetic Studies and Review of the Literature

A commercial gene panel analysis included 15 CMS causative genes: *ALG14, ALG2, CHAT, CHRNA1, CHRNB1, CHRND, CHRNE, COLQ, DOK7, DPAGT1, GFPT1, GMPPB, MUSK, RAPSN, MYO9A.*

For exome sequencing, genomic DNA extracted from peripheral blood and further captured using the Twist Human Core Exome Kit + RefSeq Panel (Twist Bioscience, San Francisco, CA) according to the manufacturer’s protocol. Paired-end (2 × 150) sequencing was performed on a NextSeq550 sequencer (Illumina). Bioinformatic analysis, including alignment to the GRCh37/hg19 human reference genome, variant and copy number variant calling and variant filtering, was done using the Varvis software (Limbus Technologies GmbH, Rostock). Targeted Sanger sequencing was subsequently performed for the index case and his parents to confirm biallelic variants in *SLC18A3*. Amplification of regions flanking the identified variants from genomic DNA was performed using two specific primer pairs (*SLC18A3*_Ex1_c315_F, 5′-CCCATAGTGCCCGACTACAT-3′ and *SLC18A3*_Ex1_c315_R, 5′-GCACGTCGTAGCTCATGC-3′;*SLC18A3*_Ex1_c1192_F, 5′-CACACCTGCAGTGGCTGTA-3′, *SLC18A3*_Ex1_c1192_R-5′GCTCAAAGCCCAGCGAGT-3′). *SLC18A3* variants are described on Refseq isoform NM_003055.3.

A review of the literature on *SLC18A3* patients was performed under the term “*SLC18A3* and congenital myasthenic syndrome”, revealing eight hits. After studying the articles, six relevant original articles and case reports were selected including all patients being reported to date [3,8,10,11,12], as well as one article describing the NME pathology in AChTKD^HOM^ mice [13]. No additional relevant articles were found in the citation lists of these articles.

### 2.2. Coherent Anti-Stokes Raman Scattering (CARS) and SHG Microscopy and Immunofluorescence

Six-micrometre-thick sections were used for the spectroscopic analysis. Sections were stored at −80 °C and under a constant nitrogen gas flow at room temperature dried for CARS and SHG measurements. No further sample preparation was applied for these studies.

The spectroscopic measurements were performed on a modified Leica TCS SP 8 CARS laser scanning microscope. A pulsed APE picoEmerald was used as laser source. We used the same measurement parameters as in our previous work [14]: briefly, for CARS measurements, the Stokes pulse was fix at 1064 nm and the pump pulse tunes to a wavelength between 804.0 nm to 826.4 nm with a step size of 0.7 nm. Both lasers were fixed at a power of 900 mW at the output port. Further subsequent laser attenuation in the Leica system was to 25%. Photomultipliertubes (PMTs) in backward and forward direction detected the generated CARS-signals in the wavelength range of 560–750 nm. With a beam splitter (560 nm), the simultaneously generated SHG signal was separated and detected with PMTs in both directions in the wavelength range of 380–560 nm. A 40× water immersion objective (IRAPO 40×/1.10 WATER) was used for CARS and SHG imaging. The measuring field was 292 × 292 µm (2048× 2048 pixel) with a pixel dwell time of approximately 10 µs. Before measuring spectra, single images were first taken with the pump pulse setting 811/814/817 nm (average of 3 images). This combination results in a CARS signal at 655/659/663 nm and corresponds to a wavenumber of 2932/2887/2841 cm^−1^. CARS spectra were acquired by tuning the pump laser from 804–826.4 nm (3039.3–2702.2 cm^−1^) with a step size of 0.7 nm (averaging of two images).

### 2.3. Statistical Evaluation of Muscle Fibre Calibers

The Leica software LAS X (ver. 2.0) was used to manually measure the length and width of fully imaged muscle fibres from the CARS single images at 2932 cm^−1^. The muscle fibre calibre was averaged from the two lengths. For muscle fibres in longitudinal section, the width of the fibre was determined as the calibre. A total of 255 fibres were analysed for the *SLC18A3*-patient. As reference, 1457 fibres were analysed from five control samples.

### 2.4. Extraction of Spectra for the Determined Features

The spectral CARS measurements were manually screened for conspicuous features. These were grouped according to their appearance (see results section) CARS spectra were extracted for the area of these features as well as for inconspicuous areas neighbouring these features, which were considered as normal reference. All spectra per area were normalized to one and subsequently averaged. A total of 283 spectra were used presented in the results section: A: 85, B: 80, C: 24, D: 54, reference: 40.

### 2.5. Fluorescent Labelling of Cryo-Embedded Muscle Sections

Ten-micrometre-thick sections of cryo-embedded muscle biopsies were fixed with 4% PFA/PBS (pH = 7.4) for 15 min at room temperature (RT). Fixed sections were blocked with 1%BSA/1%DMSO/PBS for 1 h at RT. HCS LipidTOX™ Green Neutral Lipid Stain (Invitrogen, Cat# H34475, 1:50) for neutral lipid staining, mouse anti-human Spectrin antibody (1:100) for plasma membrane staining, and MitoTracker™ Deep Red FM (Invitrogen, Cat# M22426, 1:50) for mitochondria staining were diluted in blocking buffer. As a second staining cocktail HCS LipidTOX™ Green Neutral Lipid Stain (Invitrogen, Cat# H34475, 1:50) for neutral lipid staining, HCS LipidTOX™ Red Phospholipidosis Detection Reagent (Invitrogen, Cat# H34351, 1:50) for phospholipid staining, and mouse anti-human Spectrin antibody (1:100) for plasma membrane staining were diluted in blocking buffer. The sections were incubated with the respective antibody cocktails for 4h at RT in the dark, followed by washing 3 times with blocking buffer for 15 min each. Sections stained with antibody cocktail 1 were stained over night at RT with AlexaFluor555 goat anti-mouse secondary antibody (Biolegend, Cat# 405324, 1:200) while sections stained with antibody cocktail 2 were stained overnight at RT with AlexaFluor647 goat anti-mouse secondary antibody (Biolegend, Cat# 405322, 1:200) in blocking buffer. All sections were washed 3 times with blocking buffer for 15 min each and stained with DAPI (Carl Roth, Cat# 6335.1, 1 mg/mL, 1:500) in blocking buffer for 10 min at RT. Finally, all sections were washed 2 times with blocking buffer, and one time with water for 15 min each at RT and covered with fluorescence mounting medium (Agilent Technologies, Cat# S302380-2).

### 2.6. Confocal Laser Scanning Microscopy (CLSM) and Image Processing of Fluorescent-Labelled Muscle Sections

For high-resolution microscopy of fluorescent-labelled muscle sections a Leica TCS SP8 confocal laser scanning microscope with acousto-optic tuneable filters, an acousto-optical beam splitter, internal hybrid detectors (HyD SP), and a LMT200 high precision scanning stage was used. Imaging of coverslip-embedded samples was performed via a Leica HC PL APO 63x/1.20 W CORR objective combined with digital zoom factors 1.0 or 2.0. Fluorescence signals were generated via sequential scans, exciting HCS LipidTOX™ Green Neutral Lipid Stain via an argon laser at 488 nm and detecting with an internal HyD at 500–550 nm. AlexaFluor555 counter-stained Spectrin or HCS LipidTOX™ Red Phospholipidosis Detection Reagent were excited by a diode-pumped solid-state laser at 561 nm and detected with internal HyDs at 600–650 nm. The third sequence for visualizing MitoTracker™ Deep Red FM or AlexaFluor647 counter-stained Spectrin involved a 633 nm helium-neon laser for excitation and internal HyD at 650–700 nm for detection. In the last sequential scan DAPI was excited via a 405 nm diode-pumped solid-state laser and detected by an internal HyD at 450–500 nm. Generated images were deconvoluted with Huygens Professional (SVI) and 3D-reconstructed with Imaris software (Bitplane).

## 3. Results

### 3.1. Clinical Findings

The male patient presented in this study was born prematurely at 36 + 2 weeks of gestation due to pathological cardiotocographical findings as the 2nd child of non-consanguineous healthy parents (father is a Roma originating from Poland and the mother is Syrian-Aramaic from Turkey) after an uneventful pregnancy. His older brother is clinically unaffected. Intrauterine, club feet and fetal akinesia were already diagnosed. Due to respiratory insufficiency at birth (Apgar scores 1’ 4, 5’ 4 and 10’ 6) the patient was initially mechanically ventilated over a period of 10 days. Birth measurements were within normal ranges. The patient additionally presented with arthrogryposis congenital (AMC), retrognathia, high arched palate, bilateral fluctuating ptosis, ophthalmoplegia, generalized muscular hypotonia, facial hypomimia, adducted thumbs and bulbar symptoms including feeding difficulties and weak cry. Due to respiratory insufficiency with recurrent apnoeas, cardiopulmonary reanimations with short periods of assisted ventilation were necessary during the first month post-partum. Echocardiography, electrocardiogram as well as electroencephalogram revealed normal results.

**Figure 1:**
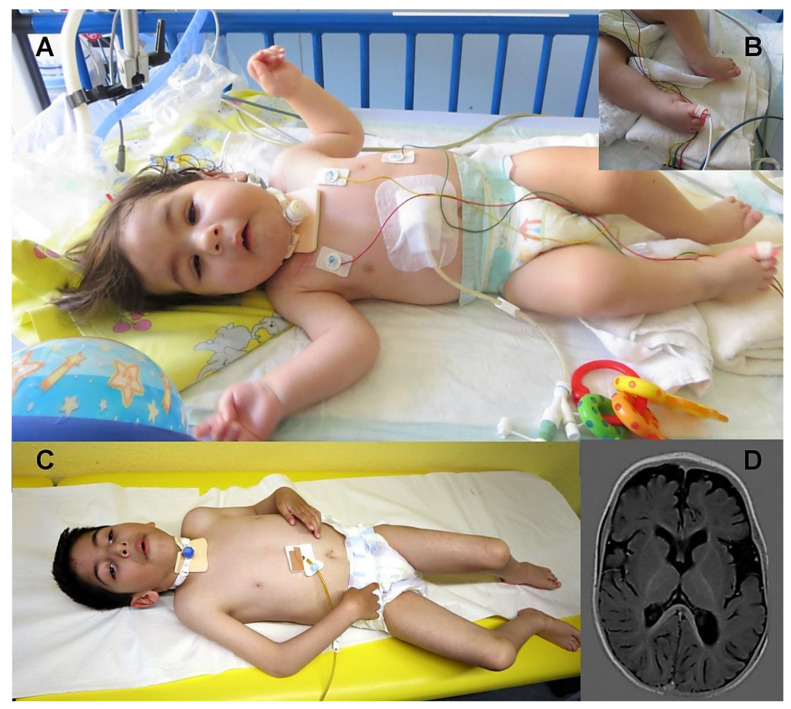
Clinical findings in the *SLC18A3* patient. (**A**) Patient at the age of 11 months displaying facial features including high arched palate (not to be seen), hypomimia, both-sided ptosis and club feet (**B**). Feeding difficulties lead to percutaneous endoscopic gastrostomy (PEG)-tube feeding, and a tracheostoma was placed due to respiratory crises with apnoea. (**C**) At the age of 5 years, the facial hypomimia and ptosis persisted. Moreover, the patient developed joint contractures in the lower extremities (knee joints) and still needed tracheostoma. (**D**) Magnetic resonance imaging (MRI) performed at the age of 11 months revealed volume reduction with bitemporal accent.

At the age of seven months, the clinical diagnosis of a CMS was established. The patient received a therapy with PS, which had a beneficial effect on ptosis and his muscle tone in extremities as well his facial hypomimia. His head control also improved, whereas bulbar and respiratory symptoms persisted. Therefore, tracheostoma was placed at the age of 11 months (Figure 1A). At the age of one year, PS therapy was paused because of uncertain efficiency and failed further motor development. However, given that his ptosis worsened, and muscular weakness decreased, PS therapy was restarted. Of note, an additional therapy with 3,4 diaminopyridine (DAP) and ephedrine was not effective. Motor development was profoundly impaired—he was able to turn himself from supine to prone position at the age of 23 months.

At last clinical follow-up at the age of 5 years 9 months, the boy still received PS therapy (dosage: 3 mg/kg/day) and was tracheostomized without assisted ventilation support. He had normal body measurements (weight on 25th percentile, length on 42nd percentile and head circumference on 33rd percentile) and was not able to sit independently but could turn himself from the prone to supine position and moved himself by rolling. His ptosis fluctuated during the day, muscular strength showed fluctuations from day to day, no diurnal fluctuations. His dysmorphic facial aspects (Figure 1 A,C) and joint contractures persisted. Due to ophthalmoplegia he could move his eyes only horizontally. His speech development appeared to be minimally impaired; he was able to pronounce single words at the age of 18 months. He visited a nursery with special medical support. A standardized intelligence testing was not performed but it was planned for him to visit a school with special support. His bulbar, muscular, and respiratory symptoms worsened during febrile or non-febrile infections. Cranial magnetic resonance imaging (MRI) showed mild generalised atrophy at the age of 11 months (Figure 1D). Repetitive stimulation at low frequency (3 Hz stimulus) showed decrement of 38% on *Nervus medianus* and 30% on *Nervus tibialis*. Because of only slight improvement under therapy with PS and due to a lack of effect of ephedrine and 3,4 DAP for the further diagnostic work-up (and to address the hypothesis of congenital myopathy), a muscle biopsy was performed at the age of three years.

### 3.2. Molecular Genetics Findings

Karyotyping and array-based comparative genomic hybridization (CGH) were initially performed and did not reveal any relevant chromosomal anomaly. Genetic testing of *SMN1* was also normal. Gene panel analysis of 15 genes involved in CMS (see Materials and Methods section) did not reveal any pathogenic variant. An intronic variant in *CHAT* (c.699-108G>T) was identified but classified aslikely benign using ACMG criteria [15].

**Figure 2:**
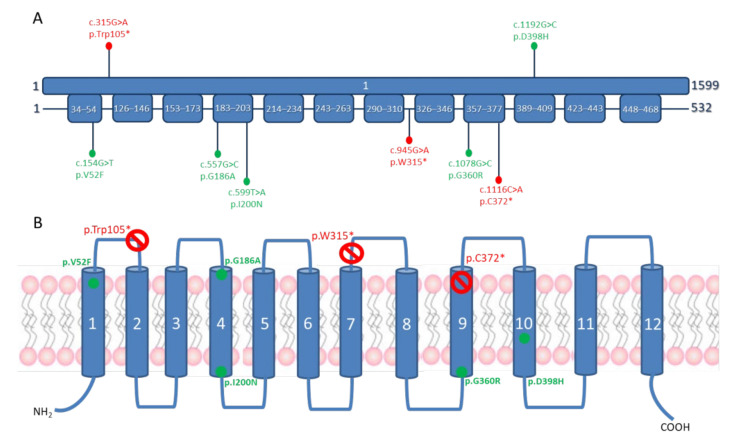
Overview of SLC18A3 pathogenic variants. (**A**) Schematic representation of SLC18A3 variants identified in this study (above) and reported in the literature (below) on the cDNA and protein. (**B**) Pathogenic variants represented on a schematic diagram of the SLC18A3 channel. Red: truncating variants; green: missense variants.

Exome sequencing in the index case revealed the presence of two heterozygous variants, c.315G>A (p.Trp105*) and c.1192G>C (p.Asp398His), in the single coding exon of *SLC18A3*. The nonsense variant (p.Trp105*) introduces an early premature termination codon at position 105, after the first transmembrane domain (Figure 2). The missense variant (p.Asp398His) replaces a highly conserved asparagine located in the 10th transmembrane domain of the VAChT by a histidine (Figure 2) and is one of the two pathogenic mutations originally reported by O’Grady et al. [3]. Sanger sequencing confirmed that both variants were each located on a different parental allele, with c.1192G>C, p.Asp398His inherited from the mother and c.315G>A, p.Trp105* inherited from the father.

### 3.3. Review of the Patients Reported in the Literature

Literature research revealed a current total of ten patients (including our patient) carrying pathogenic variants in *SLC18A3* and published clinical information, which are summarized in Table 1. In the cases of nonsense variants, both patients already died intrauterine and presented a combination of fetal akinesia and dysmorphic features (Patients 7 and 8; [8]). Two patients with homozygous missense variants had a severe neonatal presentation with feeding difficulties, respiratory problems and brain affection, and died within the first months of life or survived with severe motor and mental impairment (Patients 5 and 6; [11]). These siblings also harboured homozygous variants in *NECAB2* (p.Arg307His), which were considered to not have an influence on neuromuscular function and were thus classified as irrelevant [11]. Five patients showed muscular hypotonia, ptosis, respiratory and feeding problems already neonatal (Patients 3, 4, 9 and 10) or during infancy (Patient 2) and improvement over time with good response to PS in all. Two patients responded to additional therapy with 3,4 DAP (Patients 3 and 9). These patients harboured whole gene deletions as compound heterozygous with missense variants (Patients 2 and 4) or homozygous missense variants (Patient 3). In the case of Patient 9 with compound heterozygous missense and nonsense variants, a good response to PS was reported (can walk with support). As highlighted in Table 1, in the case of our patient, compound heterozygosity of a nonsense and missense pathogenic variants was also detected. Notably, he showed a more severe phenotype, with only ability to sit for a few minutes at the age of five, and showing only partial response to PS, although this was already started at the age of 7 months.

However, some specific phenotypic features are present in all cases: muscular hypotonia since birth, joint affection (arthrogryposis), ptosis, respiratory problems including apnoea and more severe disease course in homozygous mutations compared to compound heterozygous ones. Additionally, a brain involvement seems to be a part of the phenotype including delayed myelinisation and brain atrophy (Table 1). Fluctuation of symptoms during the day is not a prominent clinical feature, and in three patients additional nystagmus was present (Patient 3, 6 and 10).

### 3.4. Light and Electron Microscopic Studies on Quadriceps Muscle Biopsy

**Figure 3:**
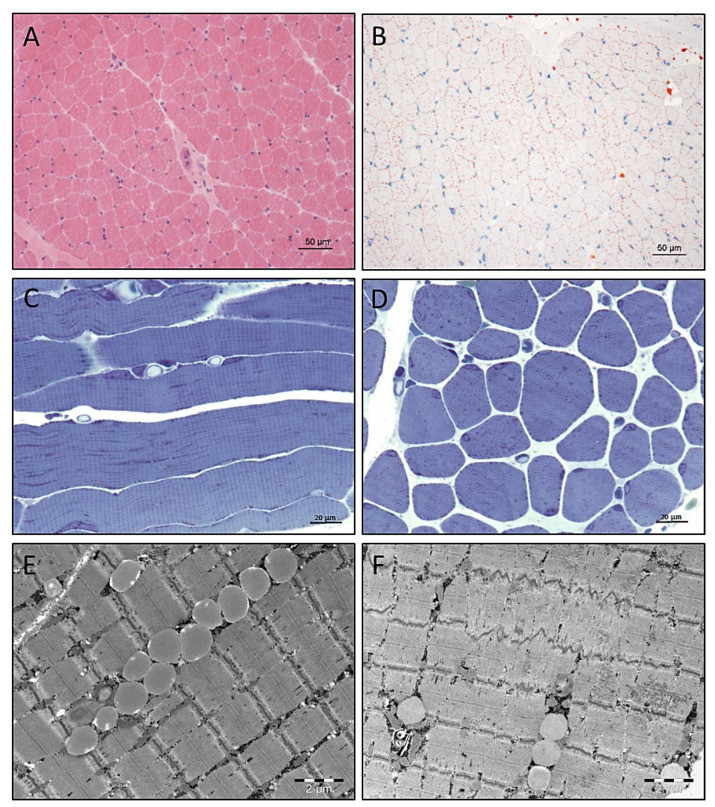
Results of histological and electron microscopic studies of quadriceps muscle derived from our *SLC18A3* patient. (**A**) Widened spectrum of muscle fibre calibres identified by H&E staining. (**B**) Increased number and size of lipid droplets in many muscle fibres identified by Oil Red O histochemistry. Scale bars = 50 µm. (**C**,**D**) Semithin resin section histology confirms the considerable widening of the calibre spectrum combined accumulation of osmiophilic material in many muscle fibres. Scale bars = 20 µm. Electron microscopic studies revealed (**E**) increased number of intermyobrillar lipid droplets, often arranged in rows and (**F**) minor focal Z-band disintegration. Scale bars = 2 µm.

Histological studies by haematoxylin and eosin (H&E) staining revealed variabilities in fibre size (Figure 3A). A profound accumulation of lipid droplets was identified by Oil Red O staining (Figure 3B). Investigation of semithin resin sections confirmed the considerable widening of the calibre spectrum combined with an accumulation of osmiophilic material in many muscle fibres (Figure 3C,D).

### 3.5. Electron Microscopy

Electron microscopic studies on the muscle biopsy derived from the *SLC18A3* patient revealed the presence of intermyofibrillar lipid droplets (Figure 3E) in addition to minor focal Z-band disintegration (Figure 3F).

### 3.6. CARS Microscopic Studies on Quadriceps Muscle Biopsy

We analysed the biochemical composition of muscle biopsies with coherent anti-Stokes Raman scattering (CARS), a non-linear variant of the Raman effect. This analysis does not require staining, markers or pre-treatment of the sample and is based on the excitation of molecular bonds associated with macromolecules, e.g., lipids or proteins. Second harmonic generation (SHG) is also a non-linear spectroscopic method that can be performed simultaneously with CARS. SHG signals indicate highly organized substances such as collagen [16]. In both methods, the signals are detected and displayed as contrast images at an excitation wavenumber.

Based on the CARS measurements, an average muscle fibre calibre of 36.5 µm ± 8.63 µm was identified for the patient. However, the five controls showed an average fibre calibre of 43.92 µm ± 23.29 µm.

**Figure 4:**
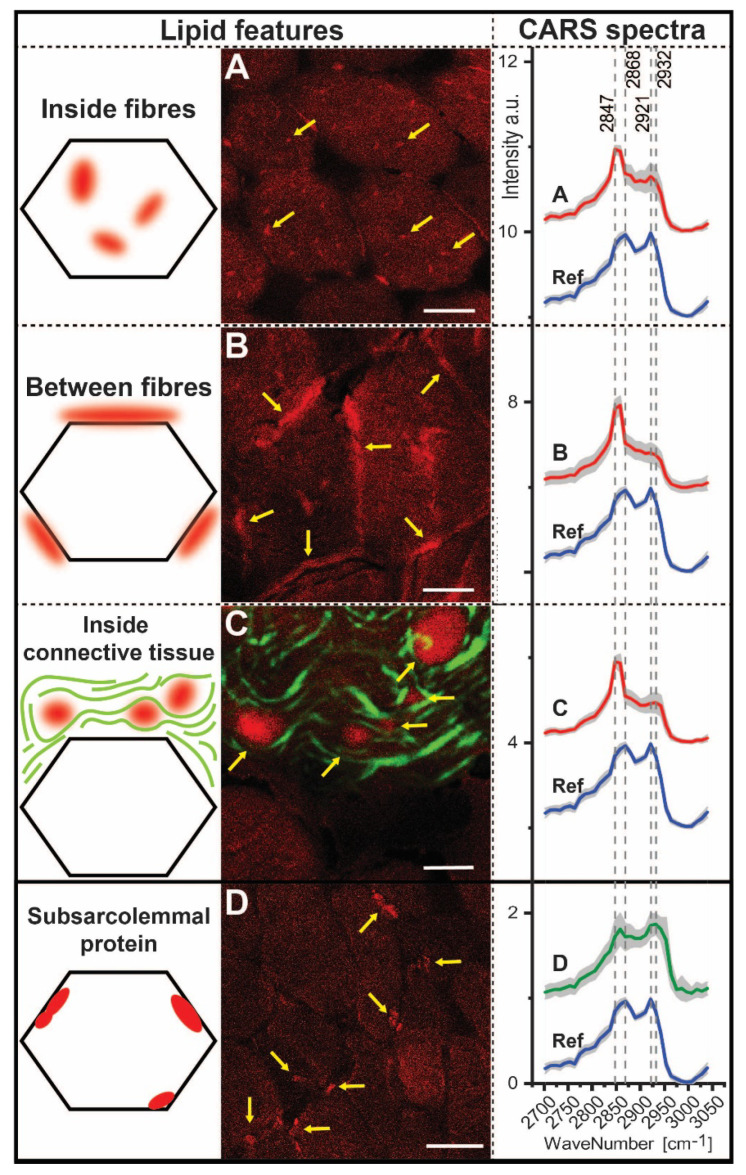
Spectroscopic findings in the muscle biopsies of the *SLC18A3* patient determined from CARS and SHG measurements. CARS images in forward direction (F-CARS) are shown in red and F-SHG images in green. CARS/SHG images showing lipid (**A**–**C**) were taken at 2847 cm^−1^; otherwise 2953 cm^−1^ (**D**). (**A**) Lipid accumulation within a muscle fibre with high F-CARS signal at 2847 cm^−1^ (marked with arrows, Scale bar: 20 µm). (**B**) Lipid accumulation between muscle fibres with high F-CARS signal at 2847 cm^−1^ (marked with arrows, 10 µm). (**C**) Lipid accumulation within connective tissue with high F-CARS signal at 2847 cm^−1^ (marked with arrows). Collagen fibres are detected with F-SHG (7.5 µm). (**D**) Subsarcolemmal protein accumulations with F-CARS signals at 2921 cm^−1^ and 2932 cm^−1^ (20 µm). A total of 283 CARS spectra were used; A: 85, B: 80, C: 24, D: 54, reference: 40.

Within the patient biopsy, we detected four spectroscopically distinct features (Figure 4). Remarkably, three of these features represent changes in lipid distribution (Figure 4A–C). In most fibres, we observed regions of increased F-CARS signal intensity at 2847 cm^−1^ (Figure 4A) and assigned the wavenumber 2847 cm^−1^ to disordered lipids [17]. Likewise, we observed regions with altered lipid homeostasis between skeletal muscle fibres (Figure 4B) and within the extracellular matrix (Figure 4C) that also displayed an increased intensity at 2847 cm^−1^ (lipid). All lipid features also show low intensity around 2921 cm^−1^ (protein), a wavenumber characteristic for protein [18,19]. Of note, we also identified subsarcolemmal protein accumulation in the *SLC18A3* patient-derived biopsy (Figure 4D), with signals within the range of 2921–2953 cm^−1^. For these protein accumulations, the intensity is lower by 2847 cm^−1^ (lipid) to the reference spectra.

A peak shift for lipids and proteins compared to the reference spectrum was identified. This peak shift can be simplified by the ratios of the intensities (Table 2). Thus, these features show a shift in the protein peak from 2921 cm^−1^ to higher wavenumbers (2932–2953 cm^−1^). The wavenumber around 2950 cm^−1^ is assigned to the asymmetric CH3 vibration. This vibration is counted among the possible vibration types of proteins [20,21,22]. We therefore assume a change in protein distribution, which is also reflected in the change in CH3 vibration. Moreover, in subsarcolemmal localized protein aggregates, protein intensities are increased compared to the reference, independent of the lipid signal. For the lipids, we also identified a shift from 2868 cm^−1^ to 2847 cm^−1^ in the feature spectra. For these features, the ratio of 2921/2868 is comparable to 2932/2968 and 2921/2847 is comparable to 2932/2847. This does not apply to the ratios of the reference. Thus, an increased signal at 2932 cm^−1^ occurs only in addition to the signal at 2921 cm^−1^.

### 3.7. Confocal Laser Scanning Microscopy (CLSM) and Image Processing of Fluorescent-Labelled Muscle Sections

**Figure 5:**
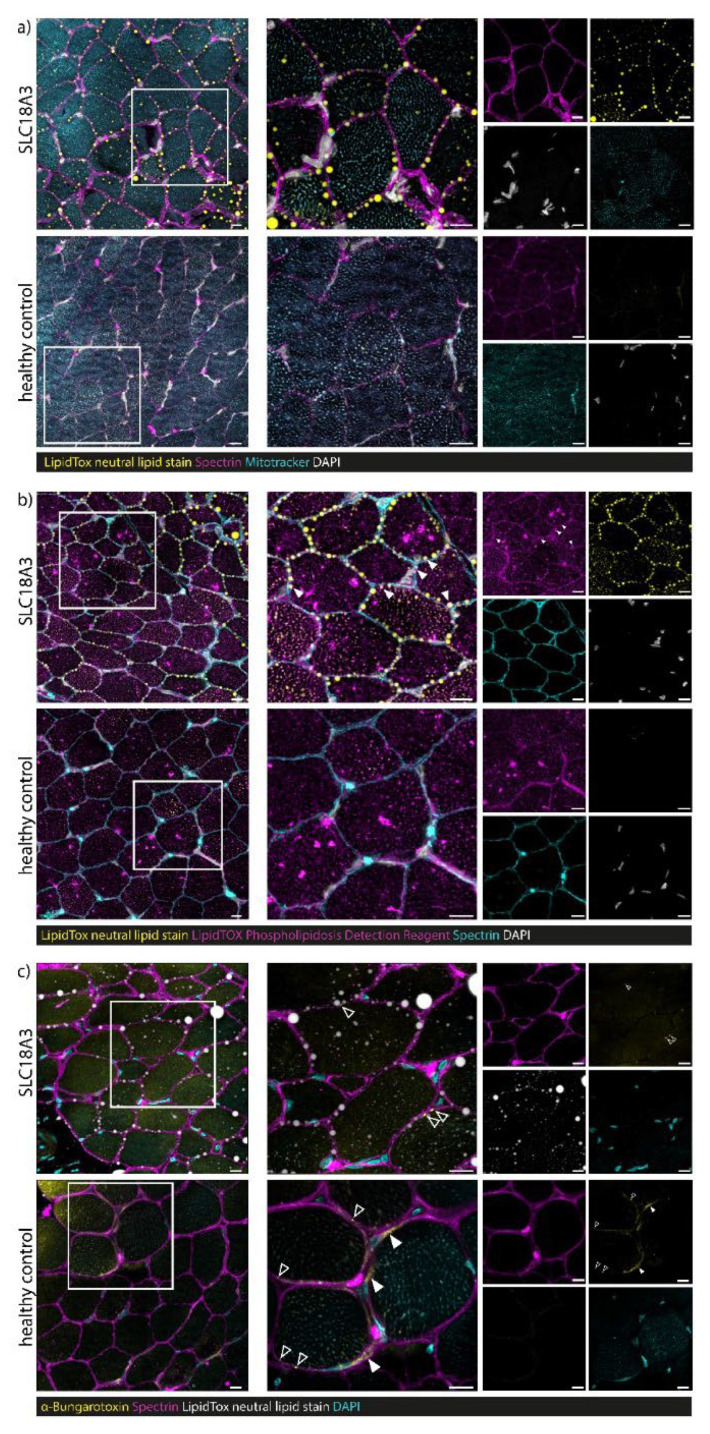
Immunofluorescence studies on muscle biopsy derived from the *SLC18A3* patient. (**a**) Staining of neutral lipids (HCS LipidTox green neutral lipid stain, yellow) and muscle membrane (Spectrin, magenta) allows the detection of lipid droplets lined up along the sarcolemma in the biopsy of our *SLC18A3* patient. Myofibre mitochondria (Mitotracker staining, turquoise) shows lower neutral lipid contents than the sarcolemma. No sarcolemma-associated lipid droplets were observed in healthy control samples. (**b**) The *SLC18A3* patient’s sarcolemma-associated neutral lipid droplets (HCS LipidTox green neutral lipid stain, yellow) are encapsulated in phospholipid membranes (HCS LipidTOX Red Phospholipidosis Detection Reagent, magenta, indicated via white arrowheads) but negative for spectrin (turquoise). No sarcolemma-associated neutral lipid droplets encapsulated by phospholipid membranes were observed in healthy control samples. (**c**) Staining with α-Bungarotoxin (yellow) indicates only a few dot-like nicotinic acetylcholine receptors at neuromuscular junctions in *SLC18A3* patient biopsy (open arrowheads) compared to healthy control in which, in addition to the dot-like structures (open arrowheads), more extensive staining can also be observed (filled arrowheads). Scale bars = 10 µm.

**Figure 6:**
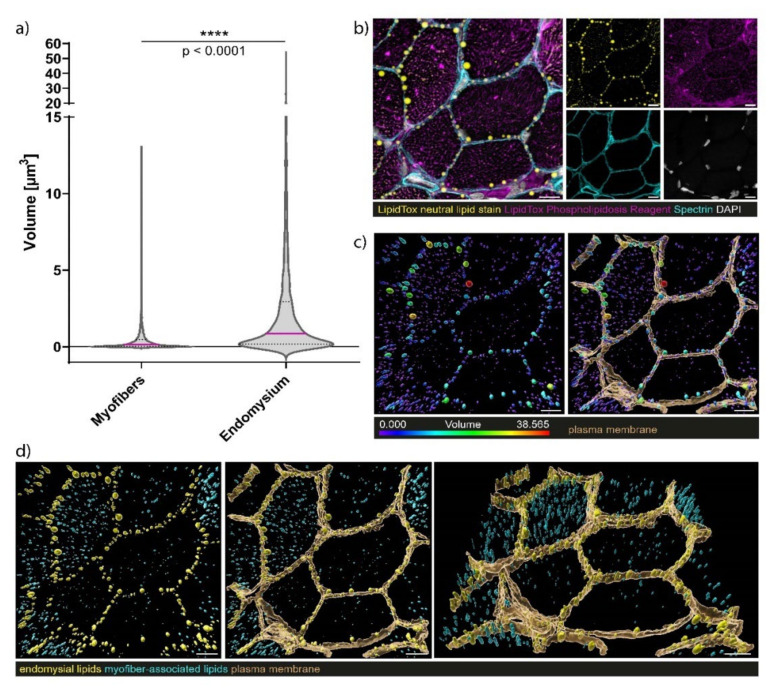
Volumetric size analysis of sarcolemma-associated lipid droplets and sarcoplasmic lipids. (**a**) Sarcolemma-associated lipid droplets have a significantly larger volume than lipid structures accumulating within the sarcoplasm (n = 6 individual field of views (FOW) from 3 independent technical replicates, n = 4664 myofiber associated lipid structures, n = 1867 sarcolemma-associated lipid droplets, two-tailed Mann-Whitney U-Test, **** *p* < 0.0001. (**b**) Exemplary FOW for volumetric lipid structure analysis showing neutral lipids (HCS LipidTox green neutral lipid stain, yellow), phospholipids (HCS LipidTOX Red Phospholipidosis Detection Reagent, magenta), sarcolemma (Spectrin, turquoise), and nuclei (DAPI, white). (**c**) Optically isolated sarcolemma is shown in brown, while lipid structures are color coded according to their volume. (**d**) Sarcolemma-associated lipids (yellow) and sarcoplasmic lipids (turquoise) are identified and separated according to their distances to the plasma membrane (brown). Scale bars = 10 µm.

Staining of neutral lipids and the sarcolemma enabled the detection of lipid droplets lined up along the sarcolemma in the biopsy of our *SLC18A3* patient. In addition, myofibre mitochondria showed lower neutral lipid contents than the sarcolemma (Figure 5). No sarcolemma-associated lipid droplets were observed in control muscle fibres. Notably, the *SLC18A3* patient’s sarcolemma-associated neutral lipid droplets are encapsulated in phospholipid membranes (but negative for Spectrin) (Figure 5). No sarcolemma-associated neutral lipid droplets encapsulated by phospholipid membranes were observed in healthy control samples. The finding of lipid droplets encapsulated by phospholipid membranes at the sarcolemmal area accords with the increased size of those compared to such within the sarcoplasm (two-tailed Mann–Whitney U-test = **** *p* < 0.0001) (Figure 6). Staining with α-Bungarotoxin revealed only a few dot-like nicotinic ACh receptors at neuromuscular junctions in the *SLC18A3* patient-derived biopsy compared to healthy control in which, in addition to the dot-like structures (open arrowheads), more extensive staining can also be observed (Figure 5C).

## 4. Discussion

### 4.1. Phenotype Associated with Bi-Allelic SLC18A3 Variants

The majority of *SLC18A3* patients already showed onset of their symptoms neonatally, only one patient during infancy, with respiratory insufficiency, bulbar symptoms or apnoea as the dominating symptoms at disease onset. Of note, *SLC18A3* patients tend to already have symptoms intrauterine and present with AMC, as was the case in our patient. Interestingly, fluctuation of the symptoms during the course of the day does not seem to be a prominent clinical feature, as is the case in most CMS mutations. Nystagmus was reported in three out of ten patients, but has not been described in other CMS cases to date. However, the *SLC18A3*-associated phenotype shares features of other known presynaptic mutations, such as *CHAT* and *SLC5A7.* In particular, the severe phenotype of *SLC5A7* patients shares clinical similarities with *SLC18A3* patients including reduced intrauterine movements with AMC and only partial response to PS in some patients [4,23]. Wang and co-workers hypothesised that profound reductions in choline transporter activity in severely affected *SLC5A7* infants and extremely low levels of mutant choline transporter at the NMJ would likely explain the extreme hypotonia, fatigability, lack of antigravity movements and respiratory insufficiency observed at birth [23]. Two *SLC18A3* patients showed brain atrophy (Patients 1 and 6), both presenting apnoea neonatally as well. In our patient, no periods of prolonged hypoxia were documented although he suffered from repeated episodes of apnoea with the need for assisted ventilation. Moreover, the only patient with later onset and no respiratory insufficiency had mental impairment (Patient 2). Our patient did not undergo standardised, age-depended intelligence testing, but his speech development and cognitive function seems to be mildly impaired. Mental impairment and brain affection in presynaptic CMS with *CHAT* and *SLC5A7* mutations seem to be based on multiple functions of ACh, the choline transporter and VAChT, not just at the NMJ, but also in central nervous system [11,23,24,25,26,27]. Along this line, decreased VAChT expression may contribute not only to neurological symptoms, but also to cognitive alterations as in the case of mice with a 70% reduction of VAChT expression [25].

The only-partial response to PS therapy could be explained by already reduced intrauterine movements causing structural muscular changes, in addition to a reduced amount of NMJs.

### 4.2. Phenotype–Genotype Correlations

In reported cases, including our patient, a genotype–phenotype correlation can be deduced: two patients with homozygous missense variants (Patients 5 and 6) presented with a severe phenotype but did not receive any therapy. Three patients with hemizygous or homozygous missense variants (Patients 2, 3, 4, 9 and 10) showed a delayed motor development, and four out of these five patients were able to walk under therapy with PS. In our patient, the combination of nonsense and missense variants in *SLC18A3* led to a more severe phenotype compared to recessive missense variants only and with poorer response to therapy with PS in comparison to these patients. His nonsense variant caused an early premature termination codon (c.315G>A; p.Trp105*), presumably leading to nonsense-mediated decay or expression of a very truncated form of the protein. However, Patients 9 and 10 also harboured compound heterozygous missense and nonsense variants. Given that the nonsense variant (c.945G>A, p.Trp315*) in these patients could lead to the expression of a less truncated protein with a residual function (compared to our patient), this might explain the manifestation of a less severe phenotype. Hakonen and co-workers postulated that this may lead to a milder phenotype in comparison to the nonsense variants, which might be caused by a residual function of the missense-mutant VAChT proteins [8]. However, systematic functional studies focussing on protein stability and activity are lacking to finally draw this conclusion. Nevertheless, the observation of intrauterine death in a *SLC18A3* patient with a homozygous nonsense variant (c.1116C>A, p.Cys372*) accords with lethality of homozygous VAChT knock-out, whereas animals with a 70% reduction of expression levels of VAChT are myasthenic and have cognitive deficits [5,28]. The latter aspect in turn suggests that minor levels of functional SLC18A3/VAChT proteins are sufficient to maintain survival associated with the manifestation of a paediatric neurological disease, thus in turn being in accordance with the hypothesis of Hakonen and co-workers [8].

Although there is a strong association between the nature of the genetic variant and the severity of the disease, one cannot exclude that other molecular factors (that have not yet been identified) might also impact the phenotypical presentation. Thus, further functional, biochemical and molecular genetic studies are crucial to systematically investigating the potential role of modifying factors (such as proteins or RNAs) that might moderate disease severity.

### 4.3. Myopathology

Interestingly, a primary vulnerability of the skeletal muscle based on histological and morphological findings was not reported for any of the nine patients reported thus far, and investigation of the muscle biopsy derived from our *SLC18A3* patient revealed variabilities in fibre size on the histological and CARS-microscopic level, suggesting a vulnerability of muscle cells upon loss of expression of functional SLC18A3/VAChT protein. Indeed, skeletal muscle of VAChT^del/del^ mutant mice showed a loss of normal myofibrillar architecture [5], a pathomorphological finding which was also observed in the biopsy of our patient on the electron microscopic level. Joviano-Santos and co-workers speculated that the affection of skeletal muscle fibres might be caused by a lack of proper ACh release in case of a mouse model with selective deletion of VAChT in motor neurons [29]. Moreover, CARS and electron microscopic studies revealed the presence of protein aggregates. This finding, along with changes of the cytoskeleton, might arise from denervation in terms of activated proteolysis in neurogenic atrophic muscle fibres [30].

Of note, our microscopic studies consistently revealed an irregular accumulation of lipid droplets between myofibrils, as well as adjacent to the sarcolemma. Thereby, droplets accumulating adjacent to the sarcolemma are larger. Further lipid staining experiments revealed that these neutral lipids are encapsulated by phospholipid membranes. Whereas the structural changes may be consequence of perturbed neuromuscular transmission as also patients harbouring *SLC5A7* mutations present with myopathic changes or generalized muscular atrophy (in one patient electron microscopic studies showed targetoid fibres), no lipid droplets were evident even if patients presented with a severe phenotype with neonatal manifestation [23], as observed in our case. Muscle pathology based on denervation–reinnervation processes at the NMJs were evident in one patient with a milder phenotype, but also did not include lipid accumulation [4]. In our study, smaller NMJs in muscle fibres derived from the *SLC18A3* patient were identified compared to such in muscle cells of a control biopsy specimen. Given that Freeman and co-workers could show that total ACh blockade in chicken embryos accompanied by reduced activation of the muscle had an adverse effect on muscle fibre differentiation and muscle fibres contained numerous large lipid droplets (similar as in our patient) [31], one might assume that proper VAChT-modulated acetylcholine transport into synaptic vesicles also has an impact on lipid homeostasis of muscle fibres (across species) during early development. 

However, more functional and biochemical studies on *SLC18A3* mutant or VAChT-depleted tissue would be necessary to unravel the exact underlying pathomechanism.

## 5. Conclusions

We report the phenotypical and myopathological findings in a *SLC18A3* patient presenting with compound heterozygous *SLC18A3* missense and nonsense variants suffering from CMS subtype 21; comparison with already published cases revealed that the combination of missense and nonsense variants possibly leads to a more severe phenotype (also affecting treatment response) than recessive missense variants only. Microscopic studies revealed fibre size variations, considerable massive accumulation of neutral lipids of different sizes (sarcolemma-associated ones were larger and encapsulated by phospholipid membranes) in addition to minor Z-band disintegrations suggesting a myopathology. However, absence of SLC18A3 expression in skeletal muscle in combination with a detrimental effect of loss of VAChT on viability in human and mice might indicate the importance of the VAChT-mediated ACh transport into synaptic vesicles in turn impacting on NMJ-integrity and lipid-homeostasis in muscle fibres.

## Figures and Tables

**Table 1:** Review of the patients with *SLC18A3* pathogenic variants reported in the literature. PS: pyridostigmine, 3,4 DAP—3,4 Diaminopyridine, n.n.—not known, n.d.—not done, m-male, f-female,mo—motnhs, MRI—magnetic resonance imaging, CT—computer tomography.

	This Study	O’Grady et al., 2016		Schwartz et al., 2017	Aran et al., 2017		Hakonen et al., 2019	Lamon et al., 2021	
	Patient 1	Patient 2	Patient 3	Patient 4	Patient 5 (sibling 1)	Patient 6 (sibling 2)	Patient 7/8 (siblings)	Patient 9	Patient 10
Causative variant	c.315G>A (p.Trp105*) & c.1192G>C (p.Asp398His); (NM_003055.2) compound heterozygous missense and nonsense	c.557G>C; (p.Gly186Ala); (NM_003055.2) hemizygous missense based on an additional 4.83MB deletion in 10q11.22-q11.23	c.1192G>C, (p.Asp398His); (NM_003055.2) homozygous missense	c.154G>T (p.Val52Phe); (NM_003055.2) hemizygous missense based on an additional 5.55MB deletion in 10q11.22-q11.23	c.1078G>A (p.Gly360Arg); (NM_003055.2) homozygous missense +NECAB2 p.R307H	c.1078G>A (p.Gly360Arg); (NM_003055.2) homozygous missense +NECAB2 p.R307H	c.1116C>A, p(Cys372*); (NM_003055.2) homozygous nonsense	c.945G>A, (p.Trp315*) & c.599T>A (p.Ile200Asn) compound heterozygous missense and nonsense	c.945G>A, (p.Trp315*) & c.599T>A, (p.Ile200Asn) compound heterozygous missense and nonsense
Age of onset	Neonatal	Infancy	Neonatal	Neonatal	Neonatal	Neonatal	Intrauterine death	Neonatal	Neonatal
Gender	Male	Male	Female	Female	Male	Male	n.n.	Male	Male
Descent	Poland/Turkey	Filipinos	Turkey	n.n.	Yemenite Jewish		Finnish	Caucasian	Caucasian
Delivery (intrauterine symptoms)	36 + 2 (reduced foetal movements)	n.n.	36 + 5	34 + 0 (reduced foetal movements)	37 weeks APGAR 4/5	40 weeks APGAR 3/3	-	38 + 1, APGAR 1/6/6	34 + 1, APGAR 7/8
Initial symptoms	Apnoeas, feeding problems, ptosis, facial hypomimia, joint contractures, muscular hypotonia	Episodes of cyanosis 2–18 months, ptosis at 1 year fatigability	Apnoeas, feeding problems, muscular hypotonia, ptosis, horizontal nystagmus, ophthalmoplegia, knee flexure contractures	Cardiorespiratory arrest, poor sucking and swallowing, amimia, ptosis	Retrognathia, axial and peripheral hypotonia, distal arthrogryposis, dislocated hips, respiratory insufficiency	Retrognathia, axial and peripheral hypotonia, distal arthrogryposis, dislocated hips, respiratory insufficiency	Fetal akinesia at 11+6/, termination of pregnancy at 15+3/12+1 weeks: arthrogryposis, partial cleft palate, dysmorphic facial features, finger abnormalities, generalised body oedema	Respiratory problems, desaturations, excessive secretion, feeding problems	Respiratory problems, apnoeas, bulbar symptoms
Additional symptoms	Undescended testis		Meconium ileus		Bilateral undescended testis, micropenis, hirsutism	Necrotising enterocolitis at the age of 2 months	-	-	-
Motor development	Delayed	Normal	Delayed		-	Severe hypotonia	-	Muscular hypotonia	Delayed
Mental development	Not tested	Partial learning deficits	Regular school	Regular school	-	Mental disability	-	Speech delay	n.n.
Follow up symptoms	Ptosis, ophthalmoplegia, facial hypomimia, swallowing problems, respiratory worsening during infections, joint contractures; can sit independently at the age of five for few minutes	Endurance problems, fatigability, ptosis, ophthalmoplegia, mild facial weakness	Walking possible at age of 4, lost of ambulance at 5, can stand, no walking	Ptosis, ophthalmoplegia, apnoeas, endurance problems, reduced walking distance	Died at the age of 5 days due to respiratory insufficiency	Ventilatory support, extreme muscular hypotonia, microcephaly, horizontal nystagmus, minimal voluntary movements in the upper extremities	-	Ptosis, “head drop”, truncal hypotonia; Speech delay, can walk with support	Muscular hypotonia, poor head control, nystagmus; no ptosis
Brain imagines	MRI: brain atrophy		MRI: mild hyperintensity of the white matter and small punctate haemorrhages in the frontal and parietal lobes	n.n.	CT: delayed myelinisation	CT: brain atrophy	-	MRI: normal	MRI: normal
Echocardiography	normal	Mild left ventricular function insufficiency	normal	n.n.	n.d.	n.d.	n.d.	n.d.	normal
Repetitive stimulation	abnormal	First normal, abnormal after exercise	abnormal	EMG normal	n.d.	n.d.	-	abnormal	n.n.
Response to therapy (start)	PS+, 3,4 DAP-, Ephedrine -	PS (14) +	PS (3 mo)+ 3,4 DAP + Distigmine + Ephedrine +	PS (neonatal)+	-	No therapy	-	PS (12 mo)+, 3,4 DAP+	PS (2 mo)+

**Table 2:** Ratio between intensities of wave numbers used for the detection of protein and lipid. The averaged spectra from Figure 4 were used to determine the ratios between wavenumbers characteristic of lipid and protein and the shifted wavenumbers.

Ratios	2932/2921	2868/2847	2932/2868	2932/2847	2921/2868	2921/2947
Inside fibres Between fibres Inside connective tissue	0.92 0.93 1.01	0.70 0.56 0.57	0.88 0.73 0.86	0.62 0.41 0.49	0.95 0.78 0.85	0.67 0.44 0.49
Subsarcolemmal protein	1.02	0.99	1.21	1.20	1.19	1.18
Reference	0.86	1.14	0.88	1.00	1.02	1.16

## Data Availability

The data that support the findings of this study are available from the corresponding author upon reasonable request.
